# Oral conditions of children with microcephaly associated with congenital Zika syndrome: a cross-sectional study

**DOI:** 10.1590/1807-3107bor-2024.vol38.0020

**Published:** 2024-03-11

**Authors:** Leni Verônica de Oliveira SILVA, José Alcides Almeida de ARRUDA, Lina Naomi HASHIZUME, Mauro Henrique Nogueira Guimarães de ABREU, Ana Cristina BORGES-OLIVEIRA

**Affiliations:** (a) Universidade Federal de Minas Gerias – UFMG, School of Dentistry, Department of Oral Surgery, Pathology, and Clinical Dentistry, Belo Horizonte, MG, Brazil.; (b) Universidade Federal do Rio Grande do Sul – UFRGS, School of Dentistry, Department of Social and Preventive Dentistry, Porto Alegre, RS, Brazil.; (c) Universidade Federal de Minas Gerias – UFMG, School of Dentistry, Department of Social and Preventive Dentistry, Belo Horizonte, MG, Brazil.

**Keywords:** Dental Care, Microcephaly, Oral Health, Mouth Mucosa, Zika Virus, Microcephaly

## Abstract

The aim of the present study was to compare the oral conditions of children with congenital Zika syndrome (CZS)-associated microcephaly, non-CZS-associated microcephaly, and normotypical children, as well as to characterize their sociodemographic aspects and medical history. A paired cross-sectional study was carried out on 14 children with CZS-associated microcephaly and 24 age-matched controls, in Belo Horizonte, in southeastern Brazil. Children’s oral conditions were assessed: dental caries experience (dmft/DMFT indices); developmental defects of enamel (DDE) index; dental anomalies; mucosal changes; lip sealing, and malocclusion (overjet, overbite, and/or posterior crossbite alterations). The quality of oral hygiene was analyzed by the simplified oral hygiene index. The children’s mothers also answered a questionnaire about sociodemographic and medical history data. The variables were analyzed descriptively. Female participants were more prevalent (60.5%), and the mean age of the participants was 4.9 years (±1.4) (range: 2–8 years) and 92.1% of their exhibited some oral condition. All participants with CZS-associated microcephaly showed absence of lip sealing and had malocclusion (100.0%). When compared to the other groups, children with CZS had a higher percentage of dental anomalies (35.7%), mucosal changes (71.4%), and unsatisfactory oral hygiene (64.3%). In a sample composed mainly of female participants aged less than 5 years, the prevalence of oral conditions and unsatisfactory oral hygiene was higher in the group with CZS-associated microcephaly, followed by the group with non-CZS-associated microcephaly. Normotypical children had the highest percentage of dental caries experience.

## Introduction

Microcephaly is a rare neurological condition involving a smaller head circumference than the standard established for an individual’s age and sex.^1,2^Multiple causes can lead to the development of this condition, including congenital infections and genetic/epigenetic changes.^3,4^ In Brazil, an outbreak of individuals born with microcephaly was associated with Zika virus infection. However, since several other systemic alterations were observed, this spectrum was recognized as congenital Zika syndrome (CZS)-associated microcephaly.^5,6^

Compelling evidence has indicated that children with CZS-associated microcephaly exhibit oral and maxillofacial alterations of unknown etiopathogenesis.^7,8^ Malocclusion, changes in palate and orofacial muscle tone, delayed tooth eruption, and dental alterations (i.e., dental anomalies and developmental defects of enamel – DDE) are among the documented disorders.^7,8^ Some oral and maxillofacial alterations may facilitate the onset or worsening of systemic complications such as dysphagia and respiratory infections. Moreover, such alterations can be accompanied by tooth sensitivity/pain which, in turn, may negatively impact the quality of life of these children.^9-11^

Although previous studies have documented cases of CZS-associated microcephaly with oral manifestations in regions considered to be the epicenter of the infection, such as in the northeast of Brazil;^7,12-14^ the literature is very scarce in other areas such as in the southeast.^15^ Furthermore, the lack of solid knowledge about the findings of rare diseases prevents progress in developing some means to reduce the burden of these conditions.^16^ In this sense, it is essential to identify and monitor affected individuals in order to minimize the associated clinical consequences and improve their quality of life.^10^

The purpose of the present study was to compare the oral conditions of children with CZS-associated microcephaly, non-CZS-associated microcephaly and normotypical children, as well as to characterize their sociodemographic aspects and medical history. The hypothesis raised was that the oral conditions of children with CZS-associated microcephaly and non-CZS-associated microcephaly would be similar and exhibit alterations regardless of Zika infection itself.

## Methodology

### Study design, setting, and ethical issues

A cross-sectional study with two comparison groups was performed between January and December 2021. Children with CZS-associated microcephaly, non-CZS-associated microcephaly, and normotypical children treated at the care unit were recruited. Efforts with the snowball sampling recruitment technique^17^ were employed to expand the number of participants; but a convenience sample was eventually used. The children from the three groups received dental care at the Faculty of Dentistry of Universidade Federal de Minas Gerais (UFMG), Belo Horizonte, Brazil.

The guidelines for Strengthening the Reporting of Observational studies in Epidemiology (STROBE) were followed.^18^ Mothers of the children willing to participate signed an informed permission or consent form and patient anonymity was ensured in conformity with the Declaration of Helsinki. This study was approved by the Human Research Ethics Committee of UFMG (process no. 38990120.0.0000.5149).

### Sample characteristics

Age zero to eight years, diagnosis of CZS-associated microcephaly or non-CZS-associated microcephaly, and normotypical development (children without special disorders such as physical/intelectual disability, syndromes, autism spectrum disorder, or acute/chronic illnesses) were set out as eligibility criteria. Participants in the different groups were matched for age. Children whose mothers refused to answer questions about any of the investigated information and/or refused to allow for a complete clinical dental examination of the child were excluded from the study. Children who did not show up for the clinical dental examination were also excluded.

### Data collection and clinical parameters

The mothers of the selected children who agreed to participate in the study were contacted by telephone, and the purpose and nature of the research were then explained and the sociodemographic data and the children’s medical history were collected. In a second phase, the children underwent oral clinical examination and received dental care at UFMG. The children are still under dental follow-up at the same place.

Initially, 30 children with microcephaly (15 CZS-associated and 15 non-CZS-associated) were invited to participate, and their mothers answered the proposed questionnaire. However, when recruited for the oral clinical examination, eight children (one CZS-associated and seven non-CZS-associated) did not show up because of maternal and child health problems and/or transport problems.

The oral clinical examination of the children was performed under artificial lighting and with appropriate personal protective equipment. Dental caries experience was determined using the decayed, missing, and filled teeth (dmft/DMFT) indices.^19^ DDE was assessed according to the modified DDE index consisting of diffuse opacity, demarcated opacity, and enamel hypoplasia.^20^ Color, shape, size, surface texture (smooth or irregular), consistency (firm or softened), and implantation and location of the mucosal changes were analyzed according to the classification of fundamental lesions proposed by Tommasi et al.^21^ and Neville et al.^22^ Dental anomalies (changes in shape, number, size, or position) were also evaluated.^22^ Clinically, children who showed any change in occlusion (overjet, overbite, and/or posterior crossbite alterations) were classified as having malocclusion.^19,23^ The absence of lip sealing (mouth posture) was assessed during the interview with the mother and the clinical exam when the children thought that they were not being observed, thus revealing the habit.^23^

The quality of oral hygiene was determined by the simplified oral hygiene index (SOHI) and was scored as follows: 0 = absence of dental plaque/dental calculus; 1 = some dental plaque/dental calculus, less than one-third of dental surface covered; 2 = dental plaque/dental calculus covering more than one-third and less than two-thirds of the dental surface; and 3 = dental plaque/dental calculus covering more than two-thirds of the dental surface. Plaque and dental calculus were evaluated separately.^24^ The final index was obtained from the sum of the codes divided by the total number of teeth examined and classified as adequate (satisfactory and fair: 0 to 2), or inadequate (deficient and poor: 2.1 to ≥ 3.1).^23,25^

### Calibration and pilot study

Before performing the oral clinical examination of the children, the examiner was trained and calibrated (at two different time points) to diagnose oral alterations such as dental caries experience and dental anomalies, DDE, malocclusion, and mucosal alterations. The level of agreement determined by the Cohen’s kappa coefficient was between 0.70 and 0.89. Subsequently, a pilot study was conducted on 15 patients (five children with CZS-associated microcephaly, five children with non-CZS-associated microcephaly, and five normotypical children). The results of the pilot study indicated that no changes to the methodology were required, and the sample was included in the investigation.

### Statistical analysis

Stratified descriptive statistics were performed using SPSS Statistics for Windows, Version 24.0. Armonk, NY: IBM Corp. Central tendency and variability measures were calculated for age, and frequency was calculated for the other variables for each of the three groups analyzed.

## Results

The age of the 38 participants (three groups) ranged between two and eight years, with a mean of 4.9 (± 1.4) years. A total of 22 children (14 with CZS-associated microcephaly and eight with non-CZS-associated microcephaly) participated in the study, representing 73.3% of the predicted universe of participants with microcephaly (n=30). There were eight losses to follow-up due to health status and/or transport problems. Sixteen normotypical children also participated in the study.

Table 1 presents the sociodemographic and medical history data of the three groups of participants. Two of the 22 children with microcephaly were wearing a respirator and 14 had some type of feeding tube (a nasogastric tube in three cases and a gastrointestinal tube in 11). Oral conditions were diagnosed in 35 children (92.1%).

**Table 1:** Descriptive data of the demographic and medical variables.

Variable	Groups			Total (n = 38)

	CZS* (n = 14)	Non-CZS^a^ microcephaly (n = 8)	Normotypical (n = 16)	

	n (%)	n (%)	n (%)	n (%)
Sex				
Male	8 (57.1)	-	7 (43.8)	15 (39.5)
Female	6 (42.9)	8 (100)	9 (56.2)	23 (60.5)
Age (years; mean (SD))	4.7 (±0.7)	4.6 (±2.3)	5.1 (±1.4)	4.9 (±1.4)
Skin color				
White	6 (42.9)	3 (37.5)	11 (68.7)	20 (52.6)
Brown or black	8 (57.1)	5 (62.5)	5 (31.3)	18 (47.4)
Prematurity				
Preterm	4 (28.6)	4 (50)	2 (12.5)	10 (26.3)
Full term	10 (71.4)	4 (50)	14 (87.5)	28 (73.7)
Low birth weight				
Yes	4 (28.6)	4 (50)	2 (12.5)	10 (26.3)
No	10 (71.4)	4 (50)	14 (87.5)	28 (73.7)
Respirator use				
Yes	1 (7.1)	1 (12.5)	-	2 (5.3)
No	13 (92.9)	7 (87.5)	16 (100)	36 (94.7)
Tube feeding				
Yes	10 (71.4)	4 (50)	-	14 (36.8)
No	4 (28.6)	4 (50)	16 (100)	24 (63.2)
Type of diet				
Free	-	1 (12.5)	16 (100)	17 (44.7)
Liquid	-	2 (25)	-	2 (5.3)
Paste	4 (28.6)	1 (12.5)	-	5 (13.2)
Tube-feeding formula	10 (71.4)	4 (50)	-	14 (36.8)
Surgery history				
Absent	5 (35.7)	3 (37.5)	14 (87.5)	22 (57.9)
Present	9 (64.3)	5 (62.5)	2 (12.5)	16 (42.1)
COVID-19 infection history				
Absent	11 (78.6)	6 (75)	16 (100)	33 (86.8)
Present	3 (21.4)	2 (25)	-	5 (13.2)
Oral conditions				
Absent	-	-	3 (18.8)	3 (7.9)
Present	14 (100)	8 (100)	13 (81.2)	35 (92.1)

^*^Congenital Zika syndrome.

The prevalence of oral conditions and inadequate oral hygiene was higher in the groups with CZS- and non-CZS-associated microcephaly when compared to the group of normotypical participants (Table 2). All individuals with CZS-associated microcephaly showed absence of lip sealing and presence of malocclusion (100.0%), followed by the group with non-CZS-associated microcephaly (absence of lip sealing = 50.0% and malocclusion = 75.0%). When compared to the other groups, children with CZS-associated microcephaly had a higher percentage of dental anomalies (35.7%), mucosal changes (71.4%), and unsatisfactory oral hygiene (64.3%). Normotypical children had the highest percentage of dental caries experience (50.0%).

**Table 2:** Descriptive data of the oral conditions.

Variable	Groups			Total (n = 38)

	CZS* (n = 14)	Non-CZS^a^ microcephaly (n = 8)	Normotypical (n = 16)	

	n (%)	n (%)	n (%)	n (%)
Absence lip sealing				
Absent	-	4 (50)	14 (87.5)	18 (47.4)
Present	14 (100)	4 (50)	2 (12.5)	20 (52.6)
DDE^b^				
Absent	4 (28.6)	2 (25)	10 (62.5)	16 (42.1)
Present	10 (71.4)	6 (75)	6 (37.5)	22 (57.9)
Dental anomalies				
Absent	9 (64.3)	6 (75)	15 (93.8)	29 (76.3)
Present	5 (35.7)	2 (25)	1 (6.2)	9 (23.7)
Malocclusion				
Absent	-	2 (25)	9 (56.2)	11 (28.9)
Present	14 (100)	6 (75)	7 (43.8)	27 (71.1)
Oral hygiene quality				
Unsatisfactory^c^	9 (64.3)	5 (62.5)	7 (43.8)	21 (55.3)
Satisfactory	5 (35.7)	3 (37.5)	9 (56.2)	17 (44.7)
Dental caries experience				
Absent	11 (78.6)	6 (75)	8 (50)	25 (65.8)
Present	3 (21.4)	2 (25)	8 (50)	13 (34.2)
Mucosal alterations				
Absent	4 (28.6)	6 (75)	16 (100)	26 (68.4)
Present	10 (71.4)	2 (25)	-	12 (31.6)

^*^Congenital Zika syndrome. ^b^ Development defects of enamel. ^c^ Unsatisfactory oral hygiene quality was considered when the children had a regular, deficient, or very poor simplified oral hygiene index (SOHI).

## Discussion

Children with congenital neurological damage are more likely to experience oral and maxillofacial alterations.^26^ A lower priority is given to the oral health of these individuals compared to their need for medical and social care; therefore, affected individuals become more vulnerable to oral diseases.^23,27^ Previous studies have also pointed out the limited access of Brazilian individuals with disabilities to oral health care services, mainly in the public health setting.^28,29^ One of the main reasons for that is the reduced number of services dedicated to the oral health care of these patients and the unpreparedness of many professionals.^23,27,28^

Oral diseases in individuals with CZS-associated microcephaly have been documented elsewhere.^12-15^ As these children grow and their stomatognathic complex develops, they show changes and they are more prone to non-communicable diseases such as gingivitis and dental caries. It is to be expected, however, that this group will have a greater demand for oral health care services in the future. Furthermore, some of the dental and maxillofacial changes exhibited by children with CZS-associated microcephaly may be linked to multiple aspects, including prematurity and low birth weight.^30^ In the present study, the percentage of enamel defects and dental anomalies, which are the most common oral alterations amongst premature and/or low birth weight individuals,^30^ was considerable among children with microcephaly. Such findings might be a confounding factor in understanding the relationship with the complications of congenital Zika infection; nevertheless, it was not possible to establish associations because of the low occurrence of these characteristics among the children assessed and the limited sample size.

Gomes et al.^31^ have suggested that the presence of microcephaly and the resulting neurological damage may represent risk factors for oral alterations not exclusively due to Zika virus infection. This is in line with the hypothesis raised in the current study, which motivated us to use a comparison group with children with microcephaly not associated with CZS. No marked differences in the frequency of alterations were observed between the groups with CZS-associated microcephaly and non-CZS-associated microcephaly. This assumption is also corroborated by the findings of former studies in which children with microcephaly due to other causes had oral alterations similar to those detected here.^26,32^

In the present study, all the children with CZS-associated microcephaly exhibited absence of lip sealing. Clinically, the absence of lip sealing may be due to factors such as malocclusion and/or hypotonia of the orofacial muscles, which, together, may contribute to the emergence or exacerbation of other complications.^33^ The recent literature suggests a possible cascade of events in individuals with neurological damage.^34,35^ Some authors believe that such alterations can cause a low tone that would trigger an inadequate lingual posture and, in some instances, dysphagia, thereby causing mouth breathing. These conditions result in malocclusion due to maxillary atresia and an ogival palate due to limitation of the adequate transverse growth of the maxilla.^34,35^

Besides malocclusion, absence of lip sealing and frequent mouth breathing can also lead to changes in the oral mucosa such as gingival hyperplasia,^36^ as detected in more than 26% of the children examined. In fact, it is difficult to pinpoint a single factor as the cause of gingival hyperplasia in children with severe neurological impairment because various medications (e.g., anticonvulsants) used by these individuals may contribute to gingival overgrowth.^37^ It is believed that there is a synergistic relationship between mouth breathing and a possible side effect of drugs, in addition to other factors such as inadequate oral hygiene.^38^ Yet, the mechanisms by which congenital Zika infection affects gingival tissues remain unclear.

The quality of oral hygiene among children with microcephaly has been mostly classified as inadequate. This status is caused by the inability and difficulty that parents/caregivers may have in performing effective oral hygiene^25^ and/or by minimizing (often due to lack of knowledge) the need for periodic toothbrushing in view of the fact that most of these children are not fed by mouth.^39^ The impossibility of oral feeding in this population is due to the severe grade of dysphagia caused by neurological impairment. This complication makes this group of individuals more prone to episodes of bronchoaspiration, with the consequent need for feeding tubes.^11,39^ In this sense, it is necessary to maintain oral hygiene even in individuals on enteral nutrition, considering the possibility of infections caused by bacterial colonization in the oral cavity with the accumulation of biofilm and dental calculus.^39^ It is important to emphasize that the implications of poor oral hygiene for children with microcephaly go beyond possible systemic infections. Notoriously, dental caries is another condition that can be caused by the accumulation of biofilm.^40^ Although in the present study the dental caries experience of the microcephaly group was low, the possibility of developing this condition cannot be ruled out.

The present study has limitations that should be taken into account. Because of its cross-sectional design and the use of a questionnaire to obtain data about the individual and medical history of the children, the study could not infer causal relationships also due to the risk of memory bias on the part of the respondents. We should also point out the difficulty in composing the microcephaly groups due to the absence of a single referral service for the health care of these affected individuals and due to unforeseen circumstances in their displacement to our service, in addition to the COVID-19 pandemic during the research period. Nonetheless, this study also has strengths that should be highlighted. First, the use of two comparison groups, one of children with non-CZS-associated microcephaly and another one with normotypical children, in order to reduce possible influences of combined characteristics on the association between independent and dependent variables. Second, the results of this study consistently provided information about the possible complications of children with CZS-associated microcephaly in a region of Brazil that had not yet been investigated. Ultimately, the reported data provide sufficient theoretical support to direct clinical practices and to reinforce the incentive for oral health care in this population. In addition, follow-up studies of children with CZS-associated microcephaly are encouraged, in order to learn about and prevent oral diseases that may arise and, in turn, improve the quality of life of those who already have oral changes.

## Conclusion

In summary, in a sample composed mainly of female participants aged less than 5 years, the prevalence of oral conditions (absence of lip sealing, malocclusion, dental anomalies, and mucosal change) and unsatisfactory oral hygiene was higher in the group with CZS, followed by the group with non-CZS-associated microcephaly. Normotypical children had the highest percentage of dental caries experience. Our hypothesis that children with CZS-associated microcephaly and non-CZS-associated microcephaly would have similar oral condition and alterations was partially confirmed by our results.
