# Erratum

**DOI:** 10.1590/1807-3107bor-2024.vol38.0020.erratum

**Published:** 2024-07-22

In article Oral conditions of children with microcephaly associated with congenital Zika syndrome: a cross-sectional study, with DOI number 10.1590/1807-3107bor-2024.vol38.0020, published in the journal Brazilian Oral Research, v. 38, e020, 2024, on

**p.1**

Where is read: José Alcides Almeida de ARRUDA

Should read: José Alcides Almeida DE ARRUDA

**p. 3, 5, 7, 9**

Where is read: de Arruda JAA

Should read: De Arruda JAA

